# Effects of onabotulinumtoxinA treatment in patients with and without allodynia: results of the COMPEL study

**DOI:** 10.1186/s10194-018-0952-1

**Published:** 2019-01-22

**Authors:** William B. Young, J. Ivan Lopez, John F. Rothrock, Amelia Orejudos, Aubrey Manack Adams, Richard B. Lipton, Andrew M. Blumenfeld

**Affiliations:** 1 Jefferson Hospital for Neuroscience, 900 Walnut Street, Second Floor, Suite #200, Philadelphia, PA 19107 USA; 2 University of South Alabama College of Medicine, Mobile, AL USA; 3 George Washington School of Medicine, Washington, DC USA; 4 Allergan plc, Irvine, CA USA; 5 Montefiore Headache Center, Department of Neurology, Department of Epidemiology and Population Health, Albert Einstein College of Medicine, Bronx, NY USA; 6 Headache Center of Southern California, The Neurology Center, Carlsbad, CA USA

**Keywords:** COMPEL, onabotulinumtoxinA, Migraine, Allodynia, Disability, Quality of life

## Abstract

**Background:**

OnabotulinumtoxinA is effective in treating chronic migraine (CM), but there are limited data assessing how allodynia affects preventive treatment responses. This subanalysis of the 108-week, multicenter, open-label COMPEL Study assessed the efficacy and safety of onabotulinumtoxinA in people with CM with and without allodynia.

**Methods:**

Patients (*n* = 715) were treated with onabotulinumtoxinA 155 U every 12 weeks for 9 treatment cycles. The Allodynia Symptom Checklist was used to identify patients with allodynia (scores ≥3). The primary outcome for this subanalysis was reduction in monthly headache days from baseline for weeks 105 to 108 in groups with and without allodynia. Other outcomes included assessments of moderate to severe headache days, disability (using the Migraine Disability Assessment [MIDAS] questionnaire), and health-related quality of life (Migraine-Specific Quality-of-Life Questionnaire [MSQ] v2). Adverse events and their relation to treatment were recorded.

**Results:**

OnabotulinumtoxinA was associated with a significant mean (SD) reduction in headache day frequency at week 108 relative to baseline in patients with (*n* = 289) and without (*n* = 426) allodynia (− 10.8 [7.1] and − 12.5 [7.4], respectively; both *P* < 0.001) that was significantly greater in patients without allodynia (*P* = 0.044 between-subgroup comparison). Moderate to severe headache days were significantly reduced at week 108 in patients with and without allodynia (− 9.6 [6.9] and − 10.5 [7.2]; both *P* < 0.001); reduction was similar between groups. MIDAS scores improved significantly at week 108 (− 53.0 [50.3] and − 37.7 [53.0]; both *P* < 0.001), with a significant between-group difference in favor of those with allodynia (*P* = 0.005). Similarly, MSQ subscale scores (Role Function Preventive, Role Function Restrictive, Emotional Function) significantly improved at week 108 for patients with and without allodynia: 20.6 (21.9) and 16.9 (20.7), 28.0 (23.3) and 24.7 (22.7), and 27.6 (26.5) and 24.9 (26.1), respectively (all *P* < 0.001). OnabotulinumtoxinA was well tolerated in patients with and without allodynia.

**Conclusion:**

Results indicate that onabotulinumtoxinA is associated with reductions from baseline in multiple efficacy outcomes for up to 108 weeks whether or not allodynia is present. The allodynia group showed a smaller treatment response for reduction in headache days, but a similar or greater treatment response for improvement in other measures. No new safety concerns were identified.

**Electronic supplementary material:**

The online version of this article (10.1186/s10194-018-0952-1) contains supplementary material, which is available to authorized users.

## Introduction

Chronic migraine (CM) is a debilitating disease occurring in 1.4% to 2.2% of adults globally [1]. It is a distinct primary headache disease defined as headaches occurring on ≥15 days per month for > 3 months, with migraine features on ≥8 days per month [2]. People with CM have more allodynia and greater levels of migraine-associated disability than people with episodic migraine [3].

In clinical practice, allodynia, a common condition among people with CM [3, 4], has been associated with a reduced likelihood of a positive response to some acute treatments for migraine [5]. Data from the American Migraine Prevalence and Prevention study demonstrated that individuals with allodynia (defined as a sum score ≥ 3 on the Allodynia Symptom Checklist [ASC]) were significantly more likely to have an inadequate response to triptans, nonsteroidal anti-inflammatory drugs, opioids, and barbiturates than those without allodynia [5]. However, it remains unclear whether allodynia also influences response to preventive treatment.

The efficacy and safety of onabotulinumtoxinA for the prevention of CM was established in the double-blind placebo-controlled Phase III REsearch Evaluating Migraine Prophylaxis Therapy (PREEMPT) trials [6–8] and was further confirmed in the 32-week open-label extension phase [9]. The Chronic Migraine OnabotulinumtoxinA Prolonged Efficacy Open-Label (COMPEL) Study was designed to gather real-world evidence on the long-term management of CM by evaluating the efficacy and safety of onabotulinumtoxinA after 9 treatments (108 weeks) [10, 11]. The COMPEL Study demonstrated that treatment with onabotulinumtoxinA 155 U was associated with sustained reduction in headache day frequency and migraine-related disability in people with CM over 108 weeks [11]. The COMPEL Study also sought to determine the efficacy of onabotulinumtoxinA in specific subgroups of interest that were not specifically assessed in or were excluded from the PREEMPT clinical study program. We undertook this analysis of the COMPEL Study to compare the efficacy and safety of onabotulinumtoxinA in patients with CM with and without allodynia at baseline.

## Methods

### Primary study design

The COMPEL Study (clinicaltrials.gov identifier NCT01516892) was a multicenter, open-label, prospective study in adult patients with CM across sites in the United States, Australia, and South Korea. The methodology of the COMPEL Study has been published previously and will be only briefly reviewed here for context [10]. OnabotulinumtoxinA (BOTOX®; Allergan plc, Dublin, Ireland) 155 U, with or without concomitant stable oral preventive medication, was administered every 12 weeks for 9 treatment cycles (108 weeks) [10] using the recommended fixed-site, fixed-dose injection paradigm [12]. Adult patients aged ≥18 years with a diagnosis of CM and with stable comorbidities were eligible for inclusion in the study if they had not previously received onabotulinumtoxinA, even if they were taking a stable oral preventive treatment at baseline. We excluded patients who had severe major depressive disorder or suicidal ideation. The study received ethical approval from the institutional review board or independent ethics committee at each site, and we obtained written informed consent from all patients before study enrollment.

The primary efficacy measure was the change from baseline in headache days per 28-day period (headache frequency) at 108 weeks (after 9 treatment cycles). The secondary efficacy measures were the change from baseline in headache days at week 60 (after 5 treatment cycles) and the mean change from baseline in the 6-item Headache Impact Test (HIT-6) total score over a 4-week period at weeks 60 and 108. Exploratory outcome measures included, but were not limited to, assessment of the change from baseline in moderate to severe headache days, HIT-6 scores throughout the study, migraine-related disability as assessed by Migraine Disability Assessment questionnaire (MIDAS) scores, and health-related quality of life as assessed by Migraine-Specific Quality-of-Life Questionnaire (MSQ) v2 scores. Moderate to severe headache days were assessed via a daily diary, and HIT-6 and MIDAS scores were assessed at each clinic visit, with higher scores indicating greater disability [13]. The MSQ, which consists of 3 subscales (Role Function Preventive, Role Function Restrictive, and Emotional Function) scored on a 0 to 100 scale, with higher scores indicating greater quality of life [14], was assessed at baseline and at weeks 48, 96, and 108.

Safety and tolerability were assessed at each visit for all patients who received ≥1 onabotulinumtoxinA treatment. Patients were withdrawn from the study for safety reasons if they showed any signs of suicidal ideation or became pregnant.

### Subgroup analysis

The subgroups with and without allodynia at baseline were assessed. The ASC was used to identify those with allodynia during the 28-day screening period. We classified patients with an ASC score ≥ 3 as having allodynia and those with an ASC score < 3 as not having allodynia at baseline [15, 16].

### Statistical analysis

As previously reported, for the primary and secondary analyses, including change in headache days and change in HIT-6 scores from baseline, missing data were imputed using a modified last-observation-carried-forward method [11]. Observed data were used for the exploratory analyses reported in this manuscript in patients with and without allodynia (headache day frequency, patient-reported outcomes, and safety). Analyses were descriptive and inferential, characterizing trends associated with onabotulinumtoxinA treatment over 108 weeks. Differences from baseline for each subgroup (ie, those with and without allodynia) were determined, including only those patients who had data at baseline and at the time point being assessed. The differences between the subgroups (ie, between those with and those without allodynia at baseline) were determined using 2-sided *t* tests (alpha = 0.05).

## Results

### Patient demographics and disposition

A total of 716 patients (safety population) were enrolled in the study. Of these patients, 715 received ≥1 dose of onabotulinumtoxinA (analysis population). In the anal-ysis population, 289 patients (40.4%) had allodynia at baseline and 426 (59.6%) did not. Patient demographics at baseline were similar across subgroups, with the exception of sex (Table 1); patients with allodynia were slightly more likely to be women (86.9%) than those without allodynia (83.3%). Clinical characteristics were generally similar across subgroups, with the exception of previous preventive treatment (Table 1); patients with allodynia at baseline were more likely to have previously taken preventive treatment (84.1%) than those without allodynia (78.6%).

**Table 1:** Baseline Patient Demographics and Clinical Characteristics of Patients With and Without Allodynia

	With Allodynia (*n* = 289)	Without Allodynia (*n* = 426)
Mean (SD) age, y	42.3 (11.3)	43.5 (11.2)
Min, max	18,72	18,73
Female, n (%)	251 (86.9)	355 (83.3)
Race, n (%)		
Caucasian	240 (83.0)	341 (80.0)
Black	16 (5.5)	25 (5.9)
Asian	31 (10.7)	58 (13.6)
Native Hawaiian or other Pacific Islander	1 (0.3)	2 (0.5)
American Indian or Alaska Native	1 (0.3)	0 (0.0)
Mean (SD) BMI, kg/m^2^	27.6 (6.5)	27.3 (6.4)
Mean (SD) age of migraine onset, y	32.2 (13.1)	32.7 (14.1)
Mean (SD) time since onset of migraine, y	10.2 (10.5)	10.8 (11.3)
Family history of migraine, n (%)	179 (61.7)	270 (63.4)
Mean (SD) headache days at baseline	21.7 (4.8)	21.9 (4.9)
Mean (SD) moderate to severe headache days at baseline	17.9 (5.6)	17.8 (5.7)
Medication use at baseline, n (%)*		
Previously taken acute medications	288 (99.3)	419 (98.4)
Previously taken preventive medications	244 (84.1)	335 (78.6)

BMI body mass index

*Data were based on the safety population

Among all patients (*N* = 716), 373 (52.1%) completed the study. Of these patients, 282 had headache day data available for all 5 study visits (including baseline). The most common reasons for study discontinuation were withdrawal of consent (*n* = 92 [12.8%]), lost to follow-up (*n* = 82 [11.5%]), lack of efficacy (*n* = 25 [4.9%]), and adverse events (AEs; n = 25 [3.5%]). Of the 290 patients with allodynia, 157 (54.1%) completed the study compared with 216 (50.7%) of the 426 patients without allodynia. Of the patients with allodynia, a cumulative total of 46 patients (15.9%) discontinued after treatment 2, 85 (29.3%) after treatment 5, and 133 (45.9%) after the final treatment. Of the patients without allodynia, a cumulative total of 85 patients (20.0%) discontinued after treatment 2, 150 (35.2%) after treatment 5, and 210 (49.3%) after the final treatment.

### Efficacy outcomes

Overall efficacy outcomes have been previously published [11] and will be reviewed only briefly here for context. In the analysis population of 715 patients, reductions in headache days were observed from the first assessment (at week 24, after 2 treatment cycles) and continued throughout the 108-week period. By week 108 (after 9 treatment cycles), onabotulinumtoxinA significantly reduced headache frequency from a mean (SD) baseline of 22.0 (4.8) days by − 10.7 (6.4) days (*P* < 0.0001), resulting in 11.3 (7.4) headache days per 28-day period. Similarly, onabotulinumtoxinA significantly reduced HIT-6 scores from a baseline score of 64.7 (4.8) by − 6.8 (6.6) at week 60 and − 7.1 (7.2) at week 108 (*P* < 0.0001). Of the 282 patients who completed the study and had headache day data available for all 5 study visits (including baseline), a slightly greater reduction in headache frequency from baseline (− 11.8 [7.3] days) was noted with onabotulinumtoxinA treatment, resulting in a slightly lower number of headache days (9.8 [8.3] days) than in the total analysis population.

#### Patients with versus without allodynia

##### Effect on headache frequency

**Fig. 1:**
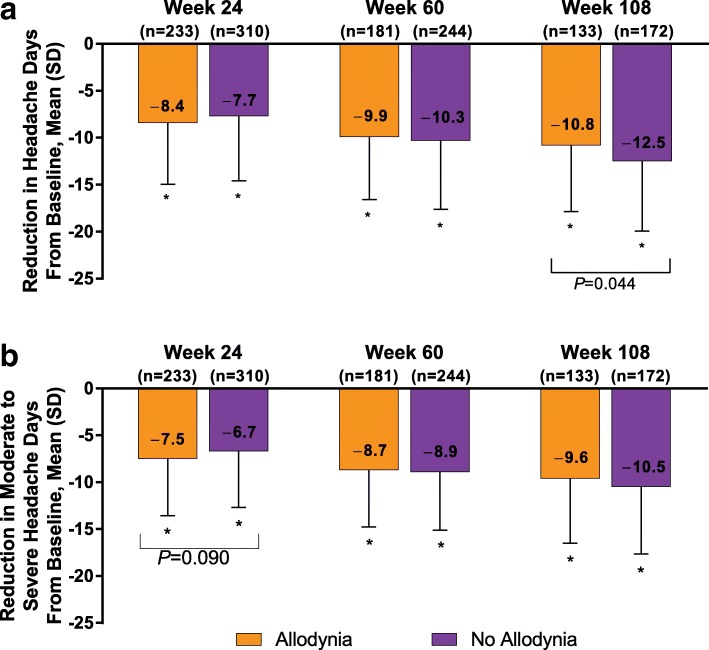
OnabotulinumtoxinA effect on **(a)** headache days and **(b)** moderate to severe headache days. *Indicates *P* < 0.001 for within-group change from baseline. *P* values shown in the figure indicate between-subgroup differences in change from baseline; data are observed data

At baseline, patients with allodynia had a mean (SD) of 21.7 [4.8] headache days compared with 21.9 [4.9] headache days in patients without allodynia. OnabotulinumtoxinA significantly reduced the mean (SD) frequency of headache days per 28-day period at week 24 in patients with and without baseline allodynia to 13.8 (8.1) and 14.3 (8.5) days, respectively; at week 60, to 11.8 (7.9) and 11.5 (8.4) days; and at week 108, to 10.5 (8.1) and 9.3 (8.3) days (*P* < 0.001 for all within-group changes from baseline; Additional file 1: Fig. S1A). The change from baseline in headache days at week 24 was − 8.4 (6.6) and − 7.7 (6.9) days, respectively; at week 60, − 9.9 (6.7) and − 10.3 (7.3) days; and at week 108, − 10.8 (7.1) and − 12.5 (7.4) days (Fig. 1a). There was no significant between-group difference in the allodynia subgroups in the mean change in headache frequency from baseline at week 24 (− 0.7 days; *P =* 0.265) and week 60 (0.4 days; *P =* 0.532); however, at week 108, there was a statistically significant difference between patients with versus without allodynia at baseline (1.7 days; *P =* 0.044) in favor of those without baseline allodynia. Similarly, onabotulinumtoxinA significantly reduced moderate to severe headache days at week 24 in patients with and without baseline allodynia to 10.7 (7.2) and 11.2 (7.6) days, respectively; at week 60, to 9.1 (6.7) and 8.6 (7.0) days; and at week 108, to 7.7 (6.8) and 7.0 (6.9) days (*P* < 0.001 for all within-group changes from baseline; Additional file 1: Figure S1B). The change from baseline in moderate to severe headache days at week 24 was − 7.5 (6.1) and − 6.7 (6.0) days, respectively; at week 60, − 8.7 (6.1) and − 8.9 (6.2) days; and at week 108, − 9.6 (6.9) and − 10.5 (7.2) days (Fig. 1b). There was no significant between-group difference in change of moderate to severe headache frequency from baseline at any time point.

##### Patient-reported outcomes

**Fig. 2:**
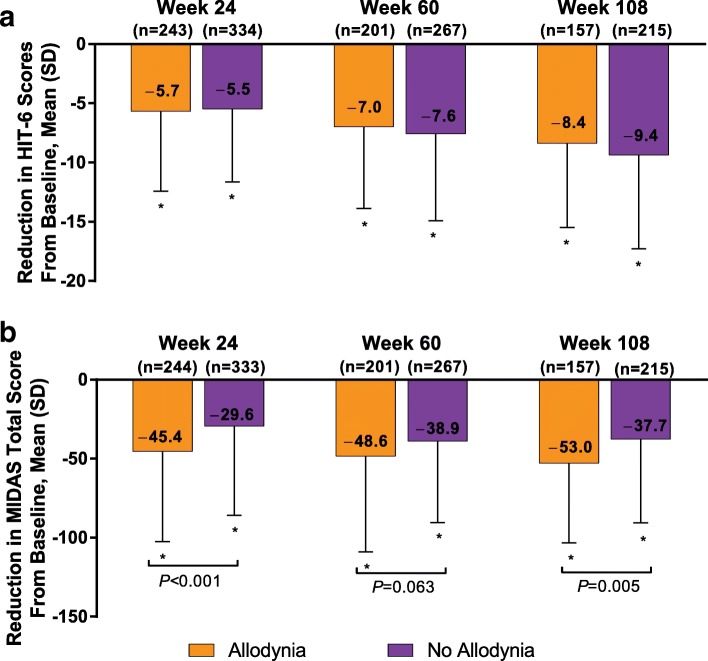
OnabotulinumtoxinA effect on **(a)** HIT-6 and **(b)** MIDAS scores. HIT-6 = 6-Item Headache Impact Test; MIDAS = Migraine Disability Assessment questionnaire. *Indicates *P* < 0.001 for within-group comparison from baseline. *P* values shown in the figure indicate between-subgroup differences in change from baseline; data are observed data

OnabotulinumtoxinA significantly reduced mean (SD) HIT-6 total scores (with higher scores indicating greater disability) from baseline scores of 65.2 (4.5) and 64.5 (5.1) in patients with and without allodynia, respectively, to 59.2 (6.9) and 58.9 (7.2) for the 28-day period before week 24; to 57.9 (6.7) and 56.6 (7.8) at week 60; and to 56.0 (6.9) and 54.5 (8.3) at week 108. The change from baseline for patients with and without allodynia increased from − 5.7 (6.7) and − 5.5 (6.2), respectively, at week 24 to − 8.4 (7.1) and − 9.4 (7.9) at week 108 (*P* < 0.001 for all within-group changes from baseline; Fig. 2a). There was no significant between-group difference in the reduction in HIT-6 scores from baseline at any time point.

OnabotulinumtoxinA significantly reduced mean (SD) MIDAS total scores (with higher scores indicating greater disability) from baseline scores of 87.4 (58.5) and 71.6 (56.2) for patients with and without allodynia, respectively, to 41.3 (47.3) and 42.3 (52.2) at week 24; 36.2 (44.6) and 29.4 (43.1) at week 60; and 22.7 (25.5) and 26.1 (43.7) at week 108 (*P* < 0.001 for all within-group changes from baseline). The change from baseline for patients with and without allodynia increased from − 45.4 (57.1) and − 29.6 (56.2), respectively, at week 24 to − 53.0 (50.3) and − 37.7 (53.0) at week 108 (Fig. 2b). There was a statistically significant between-group difference in the change of MIDAS scores from baseline in favor of those with allodynia at week 24 (mean between-group difference, − 15.8; *P* = < 0.001) and at week 108 (mean between group difference, − 15.3; *P* = 0.005).

**Fig. 3:**
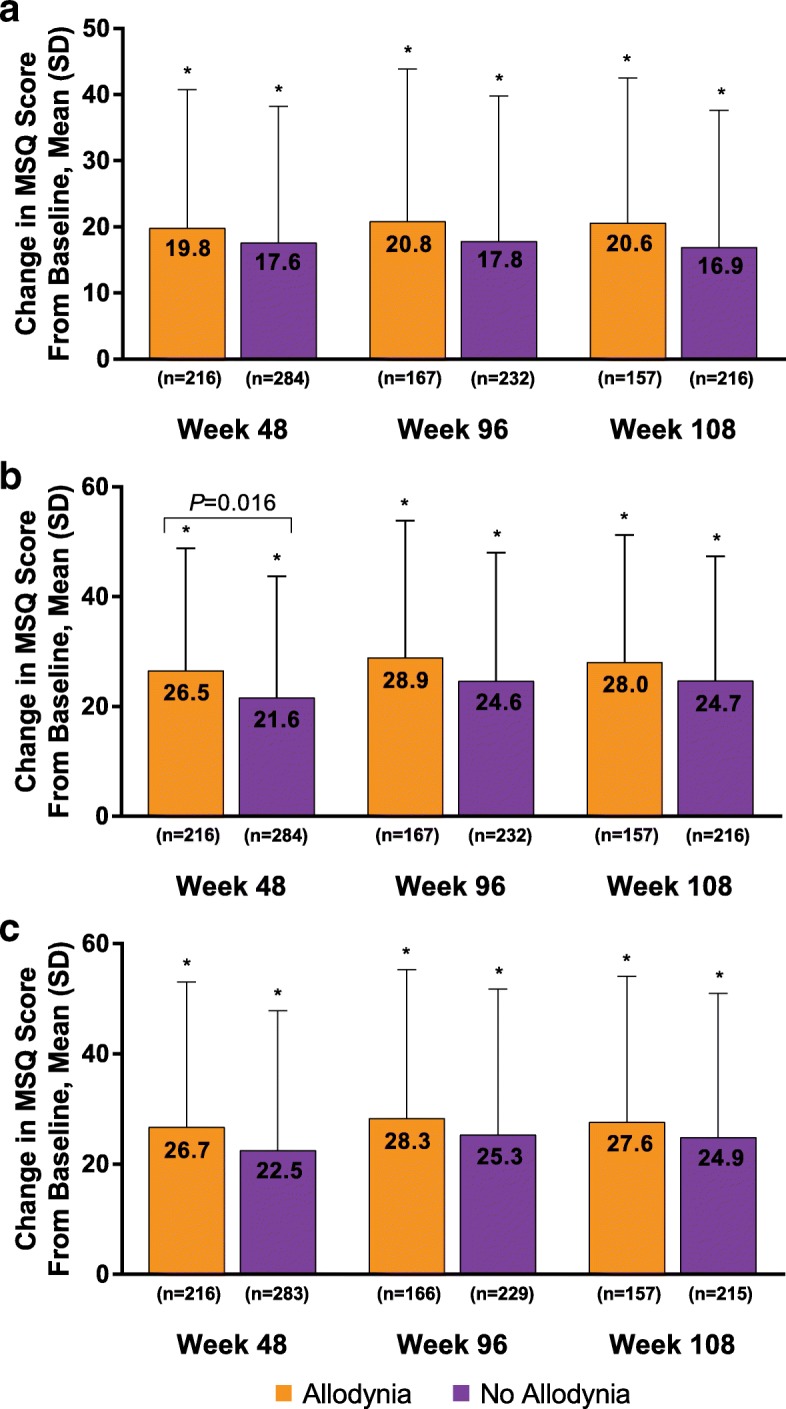
OnabotulinumtoxinA effect on MSQ **(a)** Role Function Preventive, **(b)** Role Function Restrictive, and **(c)** Emotional Function subscores. MSQ = Migraine-Specific Quality-of-Life Questionnaire. *Indicates *P* < 0.001 for within-group comparison from baseline. *P* values shown in the figure indicate between-subgroup differences in change from baseline; data are observed data

Similarly, MSQ domain scores were significantly increased (improved) at all time points compared with baseline scores regardless of allodynia status. Mean (SD) MSQ Role Function Preventive scores increased from a baseline of 57.0 (22.2) and 62.4 (22.3) for patients with and without allodynia, respectively, to 77.7 (20.4) and 80.9 (20.2) at week 48 and 81.3 (17.0) and 82.3 (19.3) at week 108 (*P* < 0.001 for all within-group changes from baseline). The change from baseline for patients with and without allodynia was similar, with no significant differences at week 48 (19.8 [20.9] and 17.6 [20.6], respectively) or at week 108 (20.6 [21.9] and 16.9 [20.7]; Fig. 3a). Mean (SD) MSQ Role Function Restrictive scores increased from a baseline of 39.2 (18.3) and 44.9 (20.1) for patients with and without allodynia, respectively, to 66.0 (21.7) and 68.3 (22.2) at week 48 and 70.2 (20.7) and 72.8 (21.2) at week 108 (*P* < 0.001 for all within-group changes from baseline). The change from baseline for patients with and without allodynia was similar at week 48 (26.5 [22.3] and 21.6 [22.1], respec-tively) and week 108 (28.0 [23.3] and 24.7 [22.7]), with no significant differences at week 108 (Fig. 3b). Mean (SD) MSQ Emotional Function scores increased from a baseline of 46.4 (25.5) and 53.1 (26.2) for patients with and without allodynia, respectively, to 75.1 (25.1) and 78.0 (22.7) at week 48 and 80.1 (20.6) and 81.7 (21.9) at week 108 (*P* < 0.001 for all within-group changes from baseline). The mean (SD) change from baseline for patients with and without allodynia was similar, with no significant differences at week 48 (26.7 [26.4] and 22.5 [25.3], respectively) or at week 108 (27.6 [26.5] and 24.9 [26.1]; Fig. 3c). The only significant between-group difference in mean (SD) change from baseline in any MSQ domain was for the change in the Role Function Restrictive domain at week 48 (4.8 [22.2]; *P* = 0.016; Fig. 3b).

### Safety and tolerability

OnabotulinumtoxinA was well tolerated in both subgroups (Table 2). AEs occurred in 185 patients (63.8%) with allodynia and 251 patients (58.9%) without allodynia. Across the safety population, serious AEs included migraine (*n* = 6 [0.8%]); suicidal ideation (*n* = 5 [0.7%]); and noncardiac chest pain, malignant melanoma, and headache (*n* = 3 [0.4%] for each), with no clear differences observed in patients with and without allodynia. Only 1 serious AE was considered to be treatment-related (generalized rash in a patient without allodynia).

**Table 2:** Summary of Adverse Events Occurring in Patients With and Without Allodynia

	With Allodynia (*n* = 290)	Without Allodynia (*n* = 426)
AEs, n (%)		
≥ 1 AE	185 (63.8)	251 (58.9)
Serious AE	36 (12.4)	39 (9.2)
Discontinuation due to AE	14 (4.8)	18 (4.2)
TRAEs, n (%)		
≥ 1 TRAE	56 (19.3)	75 (17.6)
TRAEs occurring in ≥1% of either subgroup		
Neck pain	15 (5.2)	14 (3.3)
Eyelid ptosis	6 (2.1)	12 (2.8)
Musculoskeletal stiffness	6 (2.1)	11 (2.6)
Headache	6 (2.1)	6 (1.4)
Injection site pain	5 (1.7)	9 (2.1)
Facial paresis	4 (1.4)	5 (1.2)
Muscular weakness	3 (1.0)	7 (1.6)
Migraine	3 (1.0)	4 (0.9)
Injection site hematoma	3 (1.0)	1 (0.2)
Muscle spasms	3 (1.0)	1 (0.2)
Skin tightness	1 (0.3)	6 (1.4)

AE adverse event, TRAE treatment-related adverse event

Treatment-related AEs occurring in ≥2% of either subgroup included neck pain, eyelid ptosis, musculoskeletal stiffness, injection site pain, and headache (Table 2). Thirteen patients (1.8%) in the overall population discontinued from the study as a result of treatment-related AEs.

## Discussion

The primary analysis of the COMPEL Study data demonstrated that onabotulinumtoxinA 155 U was associated with reductions in headache day frequency and a range of patient-reported outcomes in people with CM over 9 treatment cycles and 108 weeks and with a favorable tolerability profile. These results replicate and extend the findings of the PREEMPT study [6, 8, 9].

Chronic migraine in people with allodynia is typically considered difficult to manage [5], particularly with acute treatments; however, little is known about the effect of allodynia on the response to preventive treatment. The COMPEL data presented herein provide the first analyses over 9 treatment cycles of the efficacy and safety of onabotulinumtoxinA in patients with allodynia. Treatment was effective and well tolerated in patients regardless of whether they had allodynia at baseline. OnabotulinumtoxinA treatment resulted in a significantly greater improvement in MIDAS scores in patients with allodynia than in those without allodynia at baseline. Improvement in headache day frequency at week 108 was significantly lower in patients with allodynia compared with those without allodynia. For most other endpoints (eg, HIT-6 and MSQ scores), differences were not significant between those individuals with and without allodynia.

Allodynia is associated with sensitization of sensory neurons, first in the trigeminal ganglion within 10 to 20 min of the onset of migraine pain (corresponding to face and scalp pain) and then in the spinal trigeminal nucleus (also known as the trigeminal nucleus caudalis or the trigeminal cervical complex) within 60 to 120 min of the onset of pain [17, 18]. Early administration of acute treatments for migraine is reported to prevent central sensitization, but is relatively ineffective once central sensitization, as expressed by cutaneous allodynia, has occurred [19]. Across a range of drug classes routinely used for the acute treatment of migraine attacks, the presence of allodynia was associated with a greater likelihood of inadequate 2-h (ie, triptans, nonsteroidal anti-inflammatory drugs, opioids, and ergot alkaloids) and 24-h (ie, triptans, nonsteroidal anti-inflammatory drugs, opioids, and barbiturates) pain-free responses [5].

The prevalence of allodynia was lower in our study population (*n* = 289 [40.4%]) than the prevalence reported in a large group of people with migraine (63.2%) [16] and in a much smaller sample of people with CM (92.5%) [3]. Regardless, in our population, the benefits observed following preventive treatment with onabotulinumtoxinA was little influenced by the presence or absence of allodynia at baseline. After onabotulinumtoxinA treatment, there was a significant improvement in all efficacy measures at 24, 60 and 108 weeks in patients with and without allodynia compared with baseline. There was a statistically significant between-group reduction in headache frequency in favor of patients without allodynia at week 108, but not at other time points. Furthermore, there was no significant difference in the effect of onabotulinumtoxinA on change in moderate to severe headache frequency at any time point, regardless of allodynia status at baseline. These findings suggest that onabotulinumtoxinA is capable of attenuating the central sensitization of the sensory neurons associated with allodynia. However, the role of allodynia on the response to treatment needs to be explored further before definitive conclusions can be drawn. A recent meta-analysis of the treatment of migraine in people presenting to emergency departments, a population that would likely include people with allodynia, proposed that intravenous metoclopramide and prochlor-perazine, and subcutaneous sumatriptan “should be offered” to patients with acute migraine [20], suggesting that these treatments are effective, even after central sensitization of sensory neurons. Nonetheless, the availability of an effective preventive treatment will minimize the need for acute treatments in patients with CM.

Physicians and patients alike seek preventive treatment that not only reduces headache days but also improves quality of life and reduces migraine-related disability [21]. Therefore, to assess the overall effectiveness of a treatment for CM, it is recommended that in addition to efficacy measures focused on headache frequency, disease-related disability and health-related quality of life should be assessed using validated tools [22]. A > 5 point change in HIT-6 scores is considered clinically meaningful for people with migraine [23]. In our study, onabotulinumtoxinA resulted in a > 5 point change from baseline in HIT-6 total scores from week 24 onward, demonstrating a clinically meaningful improvement through week 108 in patients with or without allodynia. Similarly, MIDAS scores were reduced by approximately 40 points by week 60, regardless of allodynia status at baseline. OnabotulinumtoxinA preventive treatment did result in a slightly greater improvement in MIDAS scores in patients with allodynia than in those without allodynia, which was statistically significant at week 108 (*P* = 0.005); however, both subgroups experienced clinically meaningful improvements.

Clinically meaningful differences in MSQ domain scores vary by the specific domain being assessed (Role Function Restrictive domain, 5-point improvement; Role Function Preventive domain, 5- to 8-point improvement; Emotional Function domain, 8- to 10-point improvement) [24]. In our study, regardless of allodynia status at baseline, onabotulinumtoxinA treatment was associated with a clinically meaningful increase in all MSQ domain scores. When considered with the results from the other measures of migraine-related disability and quality of life we assessed, these results suggest that the reduction in headache frequency observed in the COMPEL Study would be clinically meaningful to patients with CM and no less so for patients with allodynia at baseline.

Others have reported that the presence of allodynia may signal a phenotype resistant to acute treatment for migraine [5, 19, 25], although the benefit of some acute treatments in patients presenting to emergency departments with migraine [20] suggests that the correlation of allodynia with treatment resistance is not clear-cut. Although the effect of onabotulinumtoxinA on headache frequency at week 108 was significantly lower in patients with allodynia than in those without allodynia, onabotulinumtoxinA reduced moderate to severe headache frequency similarly in both groups. Furthermore, onabotulinumtoxinA was at least as effective in patients with allodynia as those without allodynia across a range of secondary efficacy outcomes, suggesting that onabotulinumtoxinA is a useful preventive treatment for those patients with CM with allodynia.

### Study limitations and strengths

Open-label studies, although useful to gain additional information once the safety and efficacy profile of an intervention has been clearly established, are subject to inherent limitations, such as the lack of placebo comparison, loss to follow-up, and change in concomitant medication use over the course of the study. These limitations have been discussed more fully in a previous report [11]. Despite the low persistency rates, the treatment benefits we observed at week 24 in this anal-ysis continued to improve up to week 108, and the lack of between-group differences typically persisted, trends that reinforce our conclusions of this subgroup analysis.

In addition, fluctuations in headache frequency over time that occur with CM can make the interpretation of results difficult [26]. It is recommended that validated disease-specific tools be used to assess health-related quality of life and disability [22]. Tools such as the HIT-6 and MSQ, which were used in this study, have been validated for use in CM [27, 28], and the MIDAS questionnaire has been validated in migraine [13] and is likely to be valid for use in CM [22], including patients with CM with allodynia. Nonetheless, given the subjective reporting and open-label nature of the study, the results should be interpreted cautiously [22].

Despite the potential limitations outlined previously, the reduction in headache frequency from baseline in the overall population from the COMPEL Study at week 24 parallels results from the double-blind placebo-controlled phase of the PREEMPT study (− 7.4 days vs − 8.4 days, respectively) [8, 11]. Furthermore, results at week 24 for HIT-6 scores and week 48 for MSQ scores are similar to those reported at week 24 (HIT-6, − 4.8; Role Function Preventive, + 13.1; Role Function Restrictive, + 17.0; Emotional Function, + 17.9) after the double-blind placebo-controlled phase of the PREEMPT study [8], supporting the relevance of the results from the COMPEL Study and, by extension, this subanalysis of COMPEL data.

## Conclusions

Data from the COMPEL Study support the sustained benefit and safety of onabotulinumtoxinA for reducing headache days and disability and improving quality of life for up to 108 weeks (9 treatment cycles) in patients with CM with and without allodynia. The effect of onabotulinumtoxinA on headache days at week 108 was significantly lower in patients with allodynia than in those without allodynia. However, the effect on other efficacy measures was similar or greater in those with allodynia versus those without allodynia, despite some reports that patients with allodynia are resistant to acute treatments. No new safety concerns were identified, and onabotulinumtoxinA appeared to be well tolerated in patients with and without allodynia at baseline.

## Additional file

Additional file 1: **Figure S1.** Effect of onabotulinumtoxinA on **(A)** headache frequency and **(B)** moderate to severe headache frequency in patients with vs without allodynia at baseline. (PDF 15 kb)

## Abbreviations

AE: Adverse event
ASC: Allodynia Symptom Checklist
CM: Chronic migraine
COMPEL: Chronic migraine OnabotulinuMtoxinA Prolonged Efficacy open-Label
HIT-6: 6-item Headache Impact Test
MIDAS: Migraine Disability Assessment questionnaire
MSQ: Migraine-Specific Quality-of-Life Questionnaire v2
PREEMPT: Phase III REsearch Evaluating Migraine Prophylaxis Therapy
