# Supplementary material for: Effects of onabotulinumtoxinA treatment in patients with and without allodynia: results of the COMPEL study
Source: J Headache Pain. 2019 Jan 22;20(1):10. doi: 10.1186/s10194-018-0952-1 (PMC6734222; doi:10.1186/s10194-018-0952-1)
Supplement: Supplementary file 1 — Figure S1. Effect of onabotulinumtoxinA on (A) headache frequency and (B) moderate to severe headache frequency in patients with vs without allodynia at baseline. (PDF 15 kb) [file 10194_2018_952_MOESM1_ESM.pdf]

Supplementary Figure 1. Effect of onabotulinumtoxinA on **(A)** headache frequency and **(B)** moderate to severe headache frequency in patients with vs without allodynia at baseline.

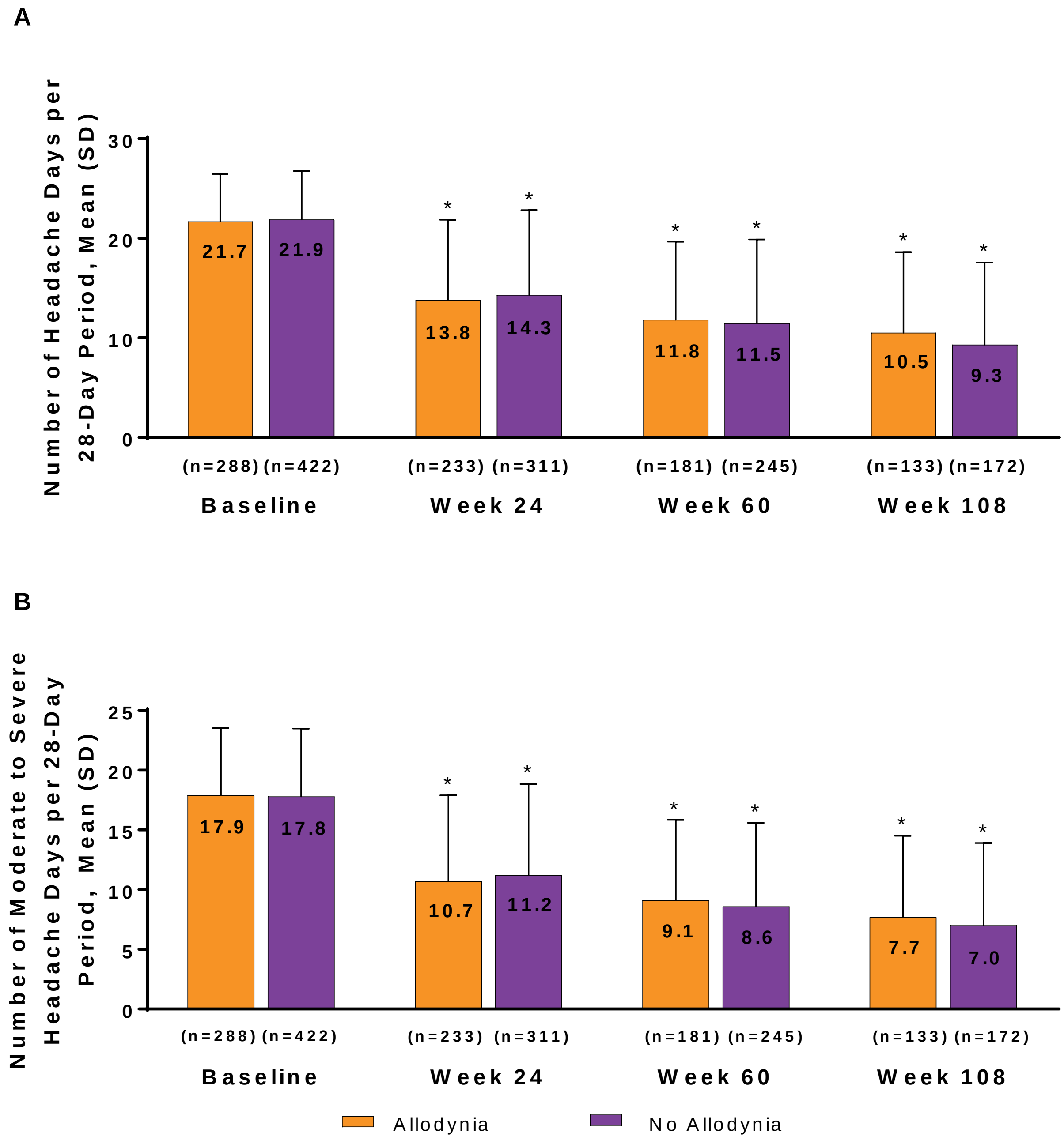

*$P<0.001$ for within-group comparison with baseline.
